# Virulence and Resistance of *Pseudomonas aeruginosa* Isolated from Poultry in Brazil

**DOI:** 10.3390/microorganisms13102402

**Published:** 2025-10-21

**Authors:** Fernanda Borges Barbosa, Maria J. Pons, Joaquim Ruiz, Yolanda Sáenz, Henrik Christensen, Terezinha Knöbl

**Affiliations:** 1 School of Veterinary Medicine and Animal Science, University of São Paulo, São Paulo 05508-270, Brazil; fernanda.borges.barbosa@usp.br; 2 Genética Molecular y Bioquímica, Universidad Científica del Sur, Lima 15067, Peru; ma.pons.cas@gmail.com (M.J.P.); joruiz.trabajo@gmail.com (J.R.); 3 Area de Microbiología Molecular, Centro de Investigación Biomédica de La Rioja (CIBIR), 26006 Logroño, Spain; ysaenz@riojasalud.es; 4 Department of Veterinary Disease Biology, Faculty of Health and Medicine, University of Copenhagen, 1870 Frederiksberg C, Denmark; hech@sund.ku.dk

**Keywords:** poultry industry, One Health, WGS, genetic diversity, *P. aeruginosa*

## Abstract

*Pseudomonas aeruginosa* is an opportunistic pathogen commonly associated with infections in hospitalized and immunocompromised patients due to its virulence and antimicrobial resistance. In the poultry industry, it has been associated with hatchery mortality. This study aimed to characterize *P. aeruginosa* isolated from pipped eggs, one-day-old chicks, and broiler carcasses obtained from a slaughterhouse in São Paulo state, Brazil. Nineteen strains of *P. aeruginosa* were selected and their virulence genes were amplified via PCR. Clonality analysis was performed using BOX-PCR, and three strains were selected for whole-genome sequencing (WGS). All isolates carried *aprA*, *plcH*, *plcN*, *lasA*, *lasB*, *lasI*, *lasR*, *rhlAB*, and *phzH*. The *exoA* gene was detected in 73.7% of strains, while *algD* was present in 21.1%. The *exoY* and *exoT* genes were present in 94.7% of strains (18/19), whereas *exoS* was present in 47.4% (9/19). None of the isolates harbored the *exoU gene*. BOX-PCR and phylogenetic analyses revealed diverse clonal patterns. The sequenced strains were classified as O3 ST116, O2 ST1649, and O3 ST1744. The presence of virulence and antimicrobial resistance determinants in poultry-associated strains underscores the need for surveillance, as these isolates may represent a source for transmission of *P. aeruginosa* to humans. Our findings highlight the importance of monitoring *P. aeruginosa* within poultry production and emphasize the value of genomic approaches to understand its diversity, evolution, and public health risks.

## 1. Introduction

*Pseudomonas* spp. are ubiquitous organisms present in soil, plants, water, animals, and insects [1]. *P. aeruginosa* is a Gram-negative pathogen which is widely distributed in the environment and is considered an opportunistic pathogen for poultry, being associated with mortality in embryos and young chickens. *P. aeruginosa* can infect both commercial and wild birds, causing dyspnea, diarrhea, pneumonia, sepsis, and death [2,3].

Pathogenic, virulent, and multidrug-resistant strains from environmental sources have been reported, establishing *P. aeruginosa* as a challenge worldwide due to its antimicrobial resistance and the presence of virulence factors, leading to nosocomial infections [4]. *P. aeruginosa* belongs to the ESKAPE (*Enterococcus faecium*, *Staphylococcus aureus*, *Klebsiella pneumoniae*, *Acinetobacter baumannii*, *Pseudomonas aeruginosa*, and *Enterobacter* species) group of pathogens with high clinical relevance in healthcare-associated infections and has been classified as a priority pathogen by the World Health Organization (WHO) [4].

The pathogenesis of *P. aeruginosa* is associated with both cell-associated and extracellular virulence factors, including flagella and pili, liposaccharides, secretion of hemolytic and nonhemolytic phospholipases C (*plcH* and *plcN*, respectively), elastases (*lasA* and *lasB*), rhamnolipids (*rhlAB*), pyocyanin biosynthesis (*phzH*), exotoxins (*exoA, exoU*, *exoY*, *exoT* and *exoS*), alkaline protease (*aprA*), and biofilm formation (*algD*) [5,6]. Most of these virulence factors are controlled by quorum sensing systems (Las, Rhl, QscR, Pqs, and Iqs), with LasIR and RhlIR being the most dominant regulatory systems [7].

*P. aeruginosa* shows intrinsic resistance to multiple classes of antimicrobial agents due to its low outer membrane permeability, the expression of efflux pumps, and the production of antibiotic-inactivating enzymes such as the AmpC cephalosporinase. *P. aeruginosa* can also acquire genes or develop chromosomal mutations that confer resistance to other agents, such as carbapenems (carbapenemases or OprD alterations), aminoglycosides (such as *aac(6*′*)-I*, or *ant*(*2*″)*-I* encoding aminoglycoside-inactivating enzymes), and quinolones (mutations in *gyrA* and *parC*) [8].

Multidrug-resistant (MDR) and extensively drug-resistant (XDR) strains have a high prevalence of association with chronic and nosocomial infections worldwide, being classified as high-risk clones. Multilocus Sequence Typing (MLST) is used to classify and analyze the diversity of strains and, at present, the MLST database (https://pubmlst.org/organisms/pseudomonas-aeruginosa (accessed on 8 April 2025)) contains 5187 different sequence types (STs). While the majority of the STs are associated with one isolate, certain STs—such as ST17, ST111, ST146, ST175, ST233, ST253, and ST277—are linked to multiple isolates from diverse countries, indicating the international distribution of these clones [9].

Although the importance of *P. aeruginosa* in human infections has been well described, reports on the diversity of *P. aeruginosa* in the poultry chain are scarce. Recently, some authors have considered the presence of *P. aeruginosa* in the poultry chain from a One Health perspective [2]. Shahat et al. (2019) described a prevalence of 20% of *Pseudomonas* species in hatcheries and poultry farms in Egypt and highlighted the selective pressure determined by the use of disinfectants, exemplified by the relation between quaternary ammonium compounds (QACs) and antibiotics [10]. In addition, Wu et al. (2023) reported the spread of MDR strains in animal-derived foods in Beijing (China) and noted a prevalence of 54.2% of *P. aeruginosa* in chicken meat, significantly higher than that in pork (19.8%) [11].

The emergence of *P. aeruginosa* in the chicken food chain has been implicated in the spread of MDR strains, as contaminated carcasses can act as reservoir of resistance genes associated with metallo-β-lactamases, AmpC, and fosfomycin [12,13]. Considering that Brazil is the world’s largest exporter of poultry meat, this study aimed to characterize *P. aeruginosa* isolated from pipped eggs, one-day-old chicks, and carcasses from São Paulo, Brazil.

## 2. Materials and Methods

### 2.1. Sample Collection and Processing

Sample collection was performed using a sterile swab from the omphalos of birds or livers of carcasses from three different batches, which were supplied by one company located in the state of São Paulo, Brazil. The samples included 45 pipped eggs (late dead in the hatchery), 74 one-day-old chicks, and 48 broiler carcasses, obtained from a slaughterhouse under the supervision of the Federal Inspection Service. The study was approved by Ethics Committee on the Use of Animals of the School of the Veterinary Medicine and Animal Science at University of São Paulo CEUA—3328060320, approval date 27 February 2023.

The refrigerated samples were sent to the Avian Medicine Laboratory of the School of Veterinary Medicine and Animal Science of the University of São Paulo (FMVZ-USP) for isolation. In the laboratory, the swabs were pre-enriched in 1% peptone water (Difco^®^, Detroit, MI, USA) and incubated at 37 °C for 18 h. This was followed by plating on MacConkey agar (Difco^®^, Detroit, MI, USA) and cetrimide selective agar (NutriSelect^®^ Plus, Darmstadt, Germany), then incubation at 37 °C for 24 h.

Large, smooth, and pigmented colonies were subjected to identification by matrix-assisted laser ionization and desorption time-of-flight mass spectrometry (MALDI-TOF MS) (Microflex^TM^–Bruker Daltonik, Bremen, Germany), using MALDI BioTyper^TM^ 3.0. Stains with scores ≥ 2.0 were kept frozen in a −80 °C freezer until further PCR analysis.

### 2.2. DNA Extraction and PCR Assay

Nineteen isolates of *P. aeruginosa* were subjected to DNA extraction by boiling at 115 °C for 20 min, then subsequently stored at −20 °C. The presence of *algD*, *plcH*, *aprA*, and *plcN* genes was detected using the primers and PCR conditions described by Pitondo-Silva and collaborators (2016) [6], while the *exoU*, *exoS*, *exoY*, *exoT*, *exoA*, *lasA*, *lasB*, *rhlAB*, *phzH*, *lasI*, and *lasR* genes were amplified using the primers and PCR conditions described by Petit and collaborators (2011) [5]. The strains PA01 and PA14 were employed as positive controls.

Clonality was evaluated using BOX-PCR typing, following the protocol described by Wolska and collaborators (2011) [14]. The BOX-PCR results were analyzed using Bionumerics v.8.1.1 (Applied Maths/bioMérieux, Kortrijk, Belgium), considering a similarity of 0.95 as a cut-off.

### 2.3. Whole-Genome Sequencing

Three isolates were selected for whole-genome sequencing (WGS) based on clonality analysis. The analysis revealed groups of highly similar strains, including one cluster containing two isolates from different sources (similarity of 100%) and another cluster with isolates from the same source (similarity of 90%). Of these, we selected the most similar isolates originating from different sources to enable comparison of their genomic characteristics across distinct environments. Two strains were isolated from pipped eggs (PAEG1 and PAEG9), while another one was isolated from a carcass (PACA1). These strains were selected due to the similarity observed in the BOX-PCR results. DNA extraction and purification were performed using the Maxwell^®^ RSC kit and automated instrument (Promega, Madison, WI, USA), according to the manufacturer’s instructions. Sequencing was performed using MiSeq (Illumina, San Diego, CA, USA). Trimming and de novo assembly were conducted using CLC genomics workbench v.23.0.4 (QIAGEN, Aarhus, Denmark). The quality of assemblies was assessed with QUAST v.5.2.0. ST, and plasmid replicons, resistance genes, and virulence genes were determined using MLST v2.0, PlasmidFinder v2.1, ResFinder v4.4.2, and VirulenceFinder v2.0 services, respectively, which are available on the website of the Center for Genomic Epidemiology (https://cge.food.dtu.dk/ (accessed on 5 February 2024)). Serotyping was determined using PAst v3 (https://github.com/Sandramses/PAst/blob/master/PAst.pl (accessed on 15 July 2023)). Molecular markers related to high-risk clones [15] were searched using BLAST (https://blast.ncbi.nlm.nih.gov/Blast.cgi?PROGRAM=blastn&PAGE_TYPE=BlastSearch&LINK_LOC=blasthome (accessed on 3 March 2025)) and Biopython v1.83.

### 2.4. Phylogenetic Analysis

A comparison was conducted between the sequenced strains and 133 publicly available *P. aeruginosa* O2 and *P. aeruginosa* O3 genomes derived from clinical human, animal, environmental, and food sources. A list of the 91 strains from GenBank and the 3 sequenced strains, containing their origin, ST, serotype, year of collection, and country, is provided in the Supplementary Materials (Supplementary Materials Table S1). The total percentage of isolates that must have a given gene for it to be considered a core gene is at least 98%. The genomes were selected from the *Pseudomonas* Genome DB (https://www.pseudomonas.com (accessed on 20 June 2023)), and only genomes with identity over 97% with respect to serotypes O2 and O3 were selected. The genomes were annotated using Prokka v1.14.6 and aligned using Roary v3.13.0. Finally, a Maximum Likelihood tree was inferred using IQ-TREE v.2.1.2, performing 1000 bootstrap replicates.

## 3. Results

Nineteen *P. aeruginosa* strains were isolated from pipped eggs (9/45), one-day-old chicks (8/74), and carcasses (2/48), with 20%, 10.8%, and 4.1% obtained from each source, respectively. All isolates were screened for virulence genes, and three representative strains were further analyzed via WGS.

### 3.1. PCR Assay

All 19 strains carried *aprA*, *plcH*, *plcN*, *lasA*, *lasB*, *lasI*, *lasR*, *rhlAB*, and *phzH*. The *exoA* gene was present in 78.9% (15/19) of isolates, while *algD* was detected in 21.1% (4/19). The type III secretion system genes were frequently observed: *exoY* and *exoT* were detected in 100% (19/19), whereas *exoS* occurred in 47.3% (9/19). None of the isolates harbored *exoU* (Table 1).

**Figure 1:**
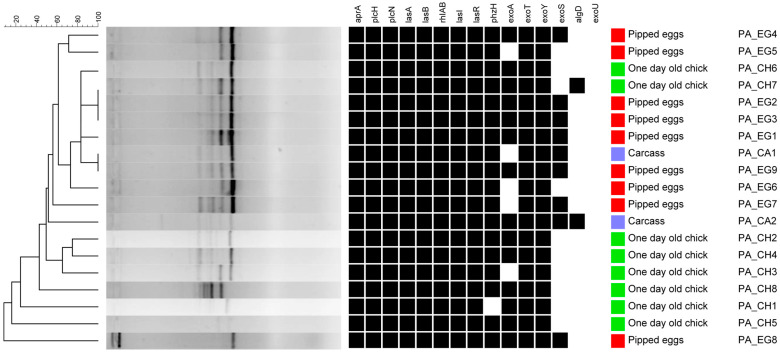
Cluster analysis of clonality (BOX-PCR) related to the distribution of virulence genes. Strains PA_CA1, PA_EG1, and PA_EG9 were subjected to WGS.

Clonality analysis showed 16 different clonal patterns, suggesting the high diversity of *P. aeruginosa* isolates in the poultry chain (Figure 1).

### 3.2. Genomic Characterization

The strains isolated from the carcass and pipped eggs subjected to WGS were classified as O3 ST116, O3 ST1744, and O2 ST1649, respectively (Table 1).

In the PubMLST and Pseudomonas Genome databases, we found 6 genomes classified as ST116, 1 genome classified as ST1649, and 2 genomes classified as ST1744 (accessed on 5 March 2025). *P. aeruginosa* ST116 has been isolated from clinical samples, soil, water, and food (tomato) [16]; while *P. aeruginosa* ST1744 has been isolated from cattle and clinical samples (sputum). On the other hand, the ST1649 strain has an unknown origin.

All sequenced isolates carried multiple resistance determinants (Table 2). The carcass-derived strain (PA_CA1) and one egg-derived strain (PA_EG9) harbored genes encoding resistance to β-lactams (*blaOXA-396*/*395*, *blaPAO*), aminoglycosides (*aph(3*′*)-IIb*), and fosfomycin (*fosA*). The other egg isolate (PA_EG1) carried resistance determinants for aminoglycosides, cephalosporins (*blaCTX-M-2*, *blaPAO*), fluoroquinolones (*crpP*), monobactams (*blaCTX-M-2*), fosfomycin (*fosA*), sulfonamides (*sul1*), and disinfectants (*qacE*).

### 3.3. Phylogenetic Analysis

**Figure 2:**
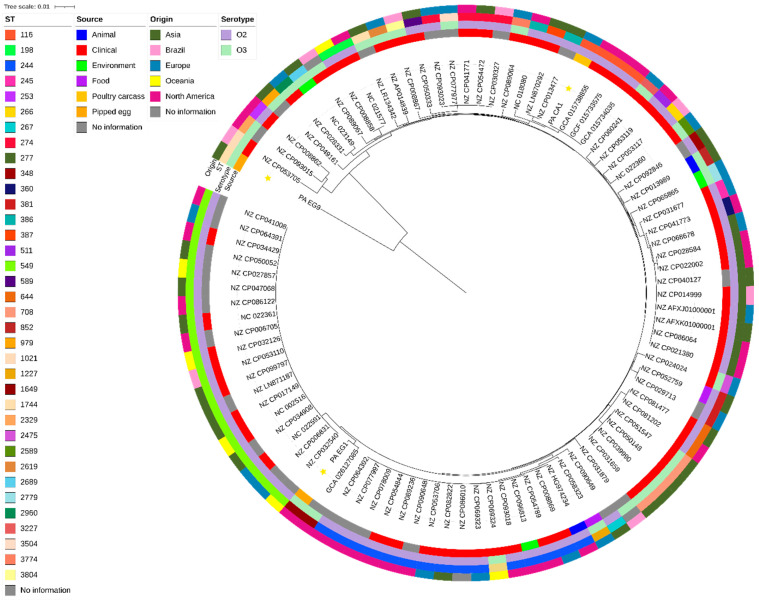
Tree rooted in the midpoint for the phylogeny of 94 genomes of O2 and O3 *Pseudomonas aeruginosa.* The sequences studied are marked with a yellow star at the tip of the branch.

Comparative analysis with 91 publicly available O2 and O3 genomes demonstrated that PA_CA1 clustered with ST116 clinical isolates from North America (GCA_015738855, GCF_015733575, GCA_015734035), suggesting possible overlap between poultry and clinical reservoirs. PA_EG1 grouped with another ST1649 strain of unreported origin, while PA_EG9 showed no close relationship with other sequenced genomes, reinforcing the genetic diversity of poultry-associated strains (Figure 2).

## 4. Discussion

This study investigated the virulence and presence of antimicrobial resistance genes in *P. aeruginosa* strains isolated from poultry farms in the state of São Paulo, Brazil. In particular, it focused on markers associated with highly virulent clones, as identified through whole-genome sequencing (WGS). The results showed an overall prevalence of 11.37%, with higher contamination in pipped eggs (20%), followed by one-day old chicks (10.8%). The contamination in carcasses was lower (4.1%), when compared to the prevalence reported by Wu et al. (2023) in Bejiing (China) [11].

Nageeb and collaborators [15] described the presence of ten molecular markers related to high-risk clones of *P. aeruginosa*. In our study, the sequenced strains had five of these molecular markers; namely, the *nfxB*, *mexZ*, *mexS*, *nalD*, and *nalC* genes. In addition, the sequenced isolates were characterized as MDR, according to definition of Magiorakos and collaborators [17], and presented mutations in *nalC*S46A, *gyrA* (T83I and D87N), and *parC* (S87W). Cabot and collaborators [18] have identified mutations in *gyrA* (T83I and D87N) and *parC* (S87W) to be involved in resistance in high-risk clones, with high-level fluorquinolone resistance having been reported.

The detection of *exoA* in most isolates is noteworthy, as this gene has been linked to severe infections in immunocompromised patients and poor clinical outcomes in hospital settings [19]. Although none of the strains harbored *exoU*, a recognized marker of hypervirulence, the high frequency of genes encoding phospholipases, proteases, elastases, and exotoxins indicate that poultry-associated isolates retain strong pathogenic potential. While the biofilm-associated gene *algD* showed a relatively low prevalence (21.1%), biofilm formation may also be associated with other determinants such as *pel*, *psl*, and c-di-GMP regulation [20]. The persistence of a pathogen may facilitate tissue damage and chronic colonization [19,21]. Mulet and collaborators (2013) described the enhanced biofilm formation ability of high-risk clones, contributing to their persistence in chronic infections [22].

The high occurrence of protease- and exotoxin-related genes underscores the ability of these isolates to degrade host tissues, induce cell lysis, and promote systemic invasion [21]. Importantly, some strains were recovered from carcasses intended for human consumption, highlighting a potential food-borne route of exposure. The observed genetic diversity likely reflects adaptation to heterogeneous environments along the poultry production chain, from hatcheries to slaughterhouses, which may contribute to the persistence of strains and variable infection outcomes.

It is important to note that while the detection of these virulence-associated genes via PCR reflects their genetic presence, it does not confirm their expression or functional activity. Further studies employing qPCR, RNA-seq, proteomic assays, and/or phenotypic analyses are necessary to determine actual gene expression levels and the functional relevance of these virulence factors. Nevertheless, our findings underscore the presence of genetically diverse *P. aeruginosa* strains carrying multiple virulence-associated genes within the poultry chain, supporting the need for ongoing surveillance.

The sequenced strains belonged to serotypes O2 and O3, which have been associated with bacteremia in human [23]. Comparative genomic analysis confirmed the presence of several determinants of virulence beyond those detected via PCR, including metalloproteases, pili, flagella, and secretion systems, supporting the pathogenic potential of these isolates. The detection of such genotypes in poultry highlights the wide environmental distribution of *P. aeruginosa* and raises concerns about reservoirs outside hospital settings [24].

In silico characterization of the resistance profile highlighted the concerning presence of antibiotic-resistant *P. aeruginosa* in poultry products. These findings are based on genomic characterization and indicate the potential for antimicrobial resistance; however, phenotypic resistance testing was not conducted in this study, which would be necessary to confirm the expression and clinical relevance of these traits. The genetic profiles observed share similarities with determinants of resistance reported in well-characterized multidrug-resistant *P. aeruginosa* strains from clinical settings and align with recent reports of resistance genes in poultry-associated isolates from other regions. In particular, the detection of disinfectant resistance genes suggests an enhanced ability to persist in sanitized environments, which could facilitate cross-contamination and increase the risk of infection in both poultry production and human exposure contexts.

Phylogenetic analysis revealed substantial diversity among the poultry isolates, with some clustering closely with clinical lineages—particularly ST116 strains from North America. This finding suggests that poultry may act as a reservoir for clinically relevant lineages, while other isolates appeared genetically distinct, pointing to parallel evolutionary trajectories within production environments. This diversity likely reflects niche-specific adaptation, antimicrobial-related selective pressures, and differences in environmental reservoirs.

*P. aeruginosa* has been inadequately explored in the considered context, considering the importance of Brazilian poultry farming in the global poultry market. This study has many limitations related to sampling, and the low number of sequenced strains does not allow for a complete picture of the country’s situation. The molecular results were also not confirmed through phenotypic testing. Therefore, further studies are needed to identify the circulation of high-risk serogroups and STs in the country. Of particular note is the identification of a strain carrying the *bla_CTX_*_-M-2_ gene, which is commonly isolated in Enterobacteriaceae and has previously been described in clinical strains of *P. aeruginosa* from Brazil [25].

Overall, the coexistence of diverse and potentially pathogenic *P. aeruginosa* lineages in the poultry chain underscores the importance of genomic monitoring. Integrating molecular epidemiology into food safety programs will be essential for understanding the evolution of strains, mitigating zoonotic risks, and limiting the spread of resistant lineages across the human–animal–environment interface.

## 5. Conclusions

This study’s findings highlight the genetic diversity within *P. aeruginosa* strains isolated from poultry products, as well as indicating their close relationship with clinical isolates. Knowledge about the genomic characteristics of these strains is crucial for comprehension of their pathogenic potential and antimicrobial resistance profiles, thus better informing measures for the control and prevention of infections. This study reinforces the need for surveillance programs in the poultry chain. The integration of public health, veterinary health, and food safety is essential to address the challenge of controlling MDR strains.

## Figures and Tables

**Table 1:** Frequency of virulence genes in Pseudomonas aeruginosa isolated from poultry, Brazil.

Virulence Genes	Samples			
	Pipped Eggs (%) *n* = 9	One-Day-Old-Chicken (%) *n* = 8	Carcass (%) *n* = 2	Total
*algD*	11.1% (1)	12.5% (1)	100% (2)	21.1% (4)
*plcH,*	100% (9)	100% (8)	100% (2)	100% (19)
*aprA*	100% (9)	100% (8)	100% (2)	100% (19)
*plcN*	100% (9)	100% (8)	100% (2)	100% (19)
*exoU*	0% (0)	0% (0)	0% (0)	0% (0)
*exoS*	77.7% (7)	0% (0)	100% (2)	47.3% (9)
*exoY*	100% (9)	100% (8)	100% (2)	100% (19)
*exoT*	100% (9)	100% (8)	100% (2)	100% (19)
*exoA*	66.6% (6)	87.5% (7)	100% (2)	78.9% (15)
*lasA*	100% (9)	100% (8)	100% (2)	100% (19)
*lasB*	100% (9)	100% (8)	100% (2)	100% (19)
*rhlAB*	100% (9)	100% (8)	100% (2)	100% (19)
*phzH*	100% (9)	100% (8)	100% (2)	100% (19)
*lasI*	100% (9)	100% (8)	100% (2)	100% (19)
*lasR*	100% (9)	100% (8)	100% (2)	100% (19)

**Table 2:** In silico characterization of sequenced strains of *P. aeruginosa* isolated from poultry.

In Silico Characterization	*P. aeruginosa* Isolated from Carcass (PA_CA1)	*P. aeruginosa* Isolated from Pipped Eggs (PA_EG1)	*P. aeruginosa* Isolated from Pipped Eggs (PA_EG9)
Serotype	O3	O2	O_3_
MLST	116	1649	1744
Plasmid	None	Col3M_1 and ColRNAI_1	None
Resistance genes	*bla_OXA-396_*, *bla_PAO_*, *aph(3*′*)-IIb*, *catB7*, and *fosA*	*bla_CTX-M-2_*, *bla_OXA-50_*, *bla_PAO_*, *aadA*, *aph(3*′*)-IIb*, *aac(3)-*VIa, *aac(6*′*)-IIc*, *cat*, *catB7*, *crpP*, *fosA*, *sul1*, and *qacE*	*bla_OXA-395_*, *bla_PAO_*, *aph(3*′*)-IIb*, *catB7*, *fosA*, and *crpP*
Virulence genes			
Phospholipases C	*plcH*, *plcN*	*plcH*, *plcN*	*plcH*
Alkaline protease	*aprA*	*aprA*	*aprA*
Rhamnolipid	*rhlAB*, *rhlI*, *rhlR*	*rhlAB*, *rhlR*	*rhlAB*, *rhlI*, *rhlR*
Mucoid related alginate	*algD*	*algD*	*algD*
Pyocyanin biosynthesis	*phzH*	*phzH*	
Type III System-Secreted effectors	*exoA*, *exoY*, *exoT*, *exoS*	*exoA*, *exoY*, *exoT*, *exoS*	*exoA*
Quorum sensing	*lasA*, *lasB*, *lasI*, *lasR*	*lasA*, *lasB*, *lasI*, *lasR*	*lasA*, *lasB*, *lasI*
GenBank accession number	SAMN47217368	SAMN47217369	SAMN47217370

## Data Availability

The whole-genome nucleotide sequence of the bacterial strains used in this work are available in the NCBI Sequence Read Archive (PRJNA1231689). The original contributions presented in this study are included in the article/Supplementary Materials. Further inquiries can be directed to the corresponding author.

## Supplementary Materials

The following supporting information can be downloaded at: https://www.mdpi.com/article/10.3390/microorganisms13102402/s1. Supplementary Table S1. Sequence, annealing temperature, amplicon size and reference of primers used to search for target genes. Supplementary Table S2. Information of publicly and sequenced genomes. References [5,6,26] are cited in the Supplementary Materials.

## References

[1] Mena K.D., Gerba C.P. (2009). Risk Assessment of *Pseudomonas aeruginosa* in water. Rev. Environ. Contam. Toxicol..

[2] Abd El-Ghany W.A. (2021). *Pseudomonas aeruginosa* infection of avian origin: Zoonosis and one health implications. Vet. World.

[3] Marouf S., Li X., Salem H.M., Ahmed Z.S., Nader S.M., Shaalan M., Awad F.H., Zhou H., Cheang T. (2023). Molecular detection of multidrug-resistant *Pseudomonas aeruginosa* of different avian sources with pathogenicity testing and in vitro evaluation of antibacterial efficacy of silver nanoparticles against multidrug-resistant *P. aeruginosa*. Poult. Sci..

[4] Santajit S., Indrawattana N. (2016). Mechanisms of Antimicrobial Resistance in ESKAPE Pathogens. Biomed. Res Int..

[5] Petit S.M., Lavenir R., Colinon-Dupuich C., Boukerb A.M., Cholley P., Bertrand X., Freney J., Doléans-Jordheim A., Nazaret S., Laurent F. (2013). Lagooning of wastewaters favors dissemination of clinically relevant *Pseudomonas aeruginosa*. Res. Microbiol..

[6] Pitondo-Silva A., Gonçalves G.B., Stehling E.G. (2016). Heavy metal resistance and virulence profile in *Pseudomonas aeruginosa* isolated from Brazilian soils. APMIS.

[7] Jesudason T. (2024). WHO publishes updated list of bacterial priority pathogens. Lancet Microbe..

[8] Bonomo R.A., Szabo D. (2006). Mechanisms of Multidrug Resistance in *Acinetobacter* Species and *Pseudomonas aeruginosa*. Clin. Infect. Dis..

[9] Oliver A., Mulet X., López-Causapé C., Juan C. (2015). The increasing threat of *Pseudomonas aeruginosa* high-risk clones. Drug Resist. Updates.

[10] Shahat H.S., Mohamed H., Abd Al-Azeem M.W., Nasef S.A. (2019). Molecular detection of some virulence genes in *Pseudomonas aeruginosa* isolated from chicken embryos and broilers with regard to disinfectant resistance. SVU-Int. J. Vet. Sci..

[11] Wu X., Yang L., Wu Y., Li H., Shao B. (2023). Spread of multidrug-resistant *Pseudomonas aeruginosa* in animal-derived foods in Beijing, China. Int. J. Food Microbiol..

[12] Rizk A.M., Elsayed M.M., Abd El Tawab A.A., Elhofy F.I., Soliman E.A., Kozytska T., Brangsch H., Sprague L.D., Neubauer H., Wareth G. (2024). Phenotypic and genotypic characterization of resistance and virulence in *Pseudomonas aeruginosa* isolated from poultry farms in Egypt using whole genome sequencing. Vet. Microbiol..

[13] Algammal A.M., Eidaroos N.H., Alfifi K.J., Alatawy M., Al-Harbi A.I., Alanazi Y.F., Ghobashy M.O., Khafagy A.R., Esawy A.M., El-Sadaa S.S. (2023). *opr*L gene sequencing, resistance patterns, virulence genes, quorum sensing and antibiotic resistance genes of XDR *Pseudomonas aeruginosa* isolated from broiler chickens. Infect. Drug Resist..

[14] Wolska K., Kot B., Jakubczak A., Rymuza K. (2011). BOX-PCR is an adequate tool for typing of clinical *Pseudomonas aeruginosa* isolates. Folia Histochem. Cytobiol..

[15] Nageeb W., Amin D.H., Mohammedsaleh Z.M., Makharita R.R. (2021). Novel Molecular Markers Linked to *Pseudomonas aeruginosa* Epidemic High-Risk Clones. Antibiotics.

[16] Bai X., Liu S., Zhao J., Cheng Y., Zhang H., Hu B., Zhang L., Shi Q., Zhang Z., Wu T. (2019). Epidemiology and molecular characterization of the antimicrobial resistance of *Pseudomonas aeruginosa* in Chinese mink infected by hemorrhagic pneumonia. Can. J. Vet. Res..

[17] Magiorakos A.P., Srinivasan A., Carey R.B., Carmeli Y., Falagas M.E., Giske C.G., Harbarth S., Hindler J.F., Kahlmeter G., Olsson-Liljequist B. (2012). Multidrug-resistant, extensively drug-resistant and pandrug-resistant bacteria: An international expert proposal for interim standard definitions for acquired resistance. Clin. Microbiol. Infection..

[18] Cabot G., Ocampo-Sosa A.A., Domínguez M.A., Gago J.F., Juan C., Tubau F., Rodríguez C., Moyà B., Peña C., Martínez-Martínez L. (2012). Genetic markers of widespread extensively drug-resistant *Pseudomonas aeruginosa* high-risk clones. Antimicrob. Agents Chemother..

[19] Michalska M., Wolf P. (2015). *Pseudomonas* Exotoxin A: Optimized by evolution for effective killing. Front. Microbiol..

[20] Liu Q., Wu Q., Liu J., Xu T., Liu J., Wu Q., Malakar P.K., Zhu Y., Zhao Y., Zhang Z. (2025). New insights into the mediation of biofilm formation by three core extracellular polysaccharide biosynthesis pathways in *Pseudomonas aeruginosa*. Int. J. Mol. Sci..

[21] Bogiel T., Prażyńska M., Kwiecińska-Piróg J., Gospodarek-Komkowska E., Mikucka A. (2021). Carbapenem-resistant *Pseudomonas aeruginosa* strains-distribution of the essential enzymatic virulence factors genes. Antibiotics.

[22] Mulet X., Cabot G., Ocampo-Sosa A.A., Domínguez M.A., Zamorano L., Juan C., Tubau F., Rodríguez C., Moyà B., Peña C. (2013). Biological markers of *Pseudomonas aeruginosa* epidemic high-risk clones. Antimicrob. Agents Chemother..

[23] Nasrin S., Hegerle N., Sen S., Nkeze J., Sen S., Permala-Booth J., Choi M., Sinclair J., Tapia M.D., Johnson J.K. (2022). Distribution of serotypes and antibiotic resistance of invasive *Pseudomonas aeruginosa* in a multi-country collection. BMC Microbiol..

[24] Klockgether J., Cramer N., Wiehlmann L., Davenport C.F., Tümmler B. (2011). *Pseudomonas aeruginosa* genomic structure and diversity. Front. Microbiol..

[25] Picão R.C., Poirel L., Gales A.C., Nordmann P. (2009). Further Identification of CTX-M-2 Extended-Spectrum β-Lactamase in *Pseudomonas aeruginosa*. Antimicrob. Agents Chemother..

[26] Li H., Li X., Wang Z., Fu Y., Ai Q., Dong Y., Yu J. (2015). Autoinducer-2 regulates Pseudomonas aeruginosa PAO1 biofilm formation and virulence production in a dose-dependent manner. BMC Microbiol..

