# Supplementary material for: Virulence and Resistance of Pseudomonas aeruginosa Isolated from Poultry in Brazil
Source: Microorganisms. 2025 Oct 21;13(10):2402. doi: 10.3390/microorganisms13102402 (PMC12566092; doi:10.3390/microorganisms13102402)
Supplement: Supplementary file 1 [file microorganisms-13-02402-s001.zip › microorganisms-3856321-supplementary.pdf]

Diversity and virulence of *Pseudomonas aeruginosa* isolated from poultry in Brazil

Supplementary Table S1. Sequence, annealing temperature, amplicon size and reference of primers used to search for target genes

Gene	Sequence	Annealing (ºC)	Amplicon (pb)	Reference
lasB	TTCTACCCGAAGGACTGATAC<br>AACACCCATGATCGCAAC	55	153	Petit *et al.,* 2013
plcN	GTTATCGCAACCAGCCCTAC<br>AGGTCGAACACCTGGAACAC	55	466	Pitondo-silva *et al,* 2016
algD	ATGCGAATCAGCATCTTTGGT<br>CTACCAGCAGATGCCCTCGGG	55	1310	Pitondo-silva *et al,* 2016
aprA	ACCCTGTCCTATTCGTTCC<br>ACCCTGTCCTATTCGTTCC	55	140	Pitondo-silva *et al,* 2016
lasA	CGCCATCCAACCTGATGCAAT<br>AGGCCGGGGTTGTACAACGGA	55	514	Petit *et al*., 2013
phzH	TTCGTTCTCGGTGGACTTCG<br>GAACTCTCGCGAAACGATGC	55	847	Li et al., 2015
plcH	GAAGCCATGGGCTACTTCAA<br>AGAGTGACGAGGAGCGGTAG	55	307	Pitondo-silva *et al,* 2016
rhlAB	TCATGGAATTGTCACAACCGC<br>ATACGGCAAAATCATGGCAAC	55	151	Petit *et al*., 2013
exoS	GCGAGGTCAGCAGAGTATCG<br>TTCGGCGTCACTGTGGAT	58	118	Petit *et al*., 2013
exoA	GACAACGCCCTCAGCATCACCAGC<br>CGCTGGCCCATTCGCTCCAGCGCT	58	396	Petit *et al*., 2013
exoU	ATGCATATCCAATCGTTG<br>TCATGTGAACTCCTTATT	58	2000	Petit *et al*., 2013
exoT	AATCGCCGTCCAACTGCATGCG<br>TGTTCGCCGAGGTACTGCTC	58	152	Petit *et al*., 2013
exoY	CGGATTCTATGGCAGGGAGG<br>GCCCTTGATGCACTCGACCA	58	289	Petit *et al*., 2013
lasI	ATGATCGTACAAATTGGTCGGC<br>GTCATGAAACCGCCAGTCG	52	605	Petit *et al*., 2013
lasR	CGGGTATCGTACTAGGTGCATCA<br>GACGGGAAAGCCAGGAAACTT	52	1100	Petit *et al*., 2013
rhlI	CTTGGTCATGATCGAATTGCTC<br>ACGGCTGACGACCTCACAC	52	730	Petit *et al*., 2013
rhlR	CAATGAGGAATGACGGAGGC<br>GCTTCAGATGAGGCCCAGC	52	625	Petit *et al*., 2013

Supplementary Table S2. Information of publicly and sequenced genomes.

ID	Origin	ST	Serotype	Origin
GCA_015734035	Clinical	116	O2	North America
GCA_015738855	Clinical	116	O2	North America
GCA_026127085	No information	1649	O3	North America
GCF_015733575	Clinical	116	O2	North America
NC_002516	Clinical	549	O2	Oceania
NC_018080	Clinical	386	O2	North America
NC_021577	Clinical	198	O2	North America
NC_022360	Clinical	2589	O3	Europe
NC_022361	Clinical	549	O2	Asia
NC_022591	Clinical	549	O2	Europe
NC_023149	Environment	2689	O3	Asia
NZ_AFXJ01000001	Clinical	277	O2	Europe
NZ_AFXK01000001	Clinical	277	O2	Asia
NZ_AP014839	Clinical	2619	O3	Europe
NZ_CP006705	No information	549	O2	North America
NZ_CP006831	No information	549	O2	Europe
NZ_CP008858	Clinical	198	O2	Oceania
NZ_CP008862	Clinical	253	O2	North America
NZ_CP008867	Clinical	3804	O3	Brazil
NZ_CP008869	Clinical	244	O2	Europe
NZ_CP013477	No information	386	O2	Asia
NZ_CP013989	Animal	852	O3	Asia
NZ_CP014999	Clinical	277	O2	Brazil
NZ_CP017149	Clinical	549	O2	Asia
NZ_CP021380	Clinical	277	O2	North America
NZ_CP022002	Clinical	277	O2	North America
NZ_CP024024	Clinical	2475	O3	Europe
NZ_CP027857	No information	549	O2	Oceania
NZ_CP028331	Clinical	2960	O3	Europe
NZ_CP028584	Clinical	277	O2	North America
NZ_CP029713	No information	381	O2	Europe
NZ_CP030327	No information	274	O2	Brazil
NZ_CP031659	Clinical	708	O3	Asia
NZ_CP031677	Clinical	245	O2	Europe
NZ_CP031879	No information	No information	O3	Brazil
NZ_CP032126	Clinical	549	O2	Oceania
NZ_CP032540	No information	549	O2	Oceania
NZ_CP034429	Clinical	549	O2	North America
NZ_CP034908	No information	549	O2	Asia
NZ_CP039990	Clinical	708	O3	Asia
NZ_CP040127	No information	277	O2	Asia
NZ_CP041008	No information	549	O2	North America
NZ_CP041771	Clinical	274	O2	North America
NZ_CP041773	Clinical	360	O2	North America
NZ_CP047068	No information	549	O2	Asia
NZ_CP049161	No information	979	O3	Asia
NZ_CP050052	No information	549	O2	Asia

NZ_CP050148	Clinical	708	O3	Asia
NZ_CP050333	Clinical	589	O2	Asia
NZ_CP051547	Clinical	708	O3	Asia
NZ_CP052759	Food	381	O2	Asia
NZ_CP053110	Clinical	549	O2	Brazil
NZ_CP053117	Clinical	266	O2	Brazil
NZ_CP053119	Clinical	511	O2	North America
NZ_CP053705	Clinical	1021	O3	Brazil
NZ_CP053706	Clinical	244	O2	Europe
NZ_CP054472	Clinical	274	O2	Asia
NZ_CP054789	Clinical	244	O2	North America
NZ_CP054844	Clinical	244	O2	North America
NZ_CP058323	Food	1227	O3	Europe
NZ_CP060241	Clinical	3227	O3	Europe
NZ_CP064391	No information	549	O2	Europe
NZ_CP064392	No information	244	O2	North America
NZ_CP065865	Environment	2779	O3	Europe
NZ_CP068678	Clinical	277	O2	Europe
NZ_CP069323	Clinical	244	O2	Europe
NZ_CP069324	Clinical	1744	O3	Oceania
NZ_CP077977	No information	3504	O3	Europe
NZ_CP077997	No information	244	O2	North America
NZ_CP078009	No information	244	O2	North America
NZ_CP081202	Clinical	644	O2	North America
NZ_CP081477	Clinical	644	O2	Asia
NZ_CP082822	Clinical	244	O2	Asia
NZ_CP086010	Clinical	244	O2	No information
NZ_CP086064	Clinical	277	O2	North America
NZ_CP086122	No information	549	O2	North America
NZ_CP089064	Clinical	3774	O3	Europe
NZ_CP089067	Clinical	No information	O3	Brazil
NZ_CP089236	Clinical	244	O2	North America
NZ_CP090648	No information	244	O2	North America
NZ_CP090649	No information	267	O2	Asia
NZ_CP092846	No information	348	O2	Asia
NZ_CP093015	No information	2329	O3	North America
NZ_CP093018	Clinical	244	O2	North America
NZ_CP093023	No information	274	O2	Europe
NZ_CP096813	Environment	244	O2	North America
NZ_CP099797	Clinical	549	O2	Asia
NZ_HG974234	Animal	244	O2	North America
NZ_LN870292	Clinical	387	O2	Asia
NZ_LN871187	No information	549	O2	Asia
NZ_LR134342	No information	1744	O3	Asia
PA_CA1	Poultry carcass	116	O2	Brazil
PA_EG1	Pipped egg	1649	O3	Brazil
PA_EG9	Pipped egg	1744	O3	Brazil
